# Heritable Differences in Schooling Behavior among Threespine Stickleback Populations Revealed by a Novel Assay

**DOI:** 10.1371/journal.pone.0018316

**Published:** 2011-03-25

**Authors:** Abigail R. Wark, Anna K. Greenwood, Elspeth M. Taylor, Kohta Yoshida, Catherine L. Peichel

**Affiliations:** 1 Division of Human Biology, Fred Hutchinson Cancer Research Center, Seattle, Washington, United States of America; 2 Program in Neurobiology and Behavior, University of Washington, Seattle, Washington, United States of America; Cajal Institute, Consejo Superior de Investigaciones Científicas, Spain

## Abstract

Identifying the proximate and ultimate mechanisms of social behavior remains a major goal of behavioral biology. In particular, the complex social interactions mediating schooling behavior have long fascinated biologists, leading to theoretical and empirical investigations that have focused on schooling as a group-level phenomenon. However, methods to examine the behavior of *individual* fish within a school are needed in order to investigate the mechanisms that underlie both the performance and the evolution of schooling behavior. We have developed a technique to quantify the schooling behavior of an individual in standardized but easily manipulated social circumstances. Using our model school assay, we show that threespine sticklebacks (*Gasterosteus aculeatus*) from alternative habitats differ in behavior when tested in identical social circumstances. Not only do marine sticklebacks show increased association with the model school relative to freshwater benthic sticklebacks, they also display a greater degree of parallel swimming with the models. Taken together, these data indicate that marine sticklebacks exhibit a stronger tendency to school than benthic sticklebacks. We demonstrate that these population-level differences in schooling tendency are heritable and are shared by individuals within a population even when they have experienced mixed-population housing conditions. Finally, we begin to explore the stimuli that elicit schooling behavior in these populations. Our data suggest that the difference in schooling tendency between marine and benthic sticklebacks is accompanied by differential preferences for social *vs.* non-social and moving *vs.* stationary shelter options. Our study thus provides novel insights into the evolution of schooling behavior, as well as a new experimental approach to investigate the genetic and neural mechanisms that underlie this complex social behavior.

## Introduction

Social groups, including schools, flocks, and swarms, are widespread in the animal kingdom. Such groups are thought to provide a number of fitness benefits for their participants, including a reduction in predation risk and an increase in foraging efficiency, among others [1]. However, there are also costs to the formation of social groups, and not all animals form congregations [1]. Although the ultimate, evolutionary explanations for this diversity have been the subject of extensive theoretical and comparative work, the proximate biological mechanisms that generate differences in the tendency to join a group between species or populations have received less attention. Investigating these mechanisms requires the development of methods that can reliably assess individual behavior within the context of a social group. In the present paper, we describe a novel method that provides a powerful new opportunity to address this issue in the context of schooling behavior in fish.

Fish schools provide a particularly dramatic example of animals behaving socially [2]. To form cohesive schools, individuals must not only gather together socially (*i.e.* shoaling) but must also precisely synchronize their position, speed, and angle of motion with the behavior of their neighbors (*i.e.* schooling) [3], [4]. The intricate social interactions that make schooling fascinating to biologists also present substantial experimental complications because the behavior of an individual can be strongly influenced by the behavior of others within the school [5]–[7]. In addition, schools are dynamic and members are likely to behave variably across trials, potentially imposing differential influences on individual experiences during testing. Thus, characterizing individual behavior within a freely behaving social group can be problematic or misleading. On the other hand, schooling behavior cannot be properly understood outside the context of a social group. Some previous investigations have circumvented the need to control for group behavior by utilizing a shoaling assay to measure preference for social affiliation [1], [8]–[15]. However, shoaling assays and other behavioral tests that physically separate the individual from the social group eliminate the possibility of measuring spacing, parallel alignment, and synchronous movement. Therefore, these assays cannot accurately assess schooling as they fail to measure key aspects of the behavior.

To overcome these challenges, we developed the “model school assay”. This assay uses an artificial school of model fish to evaluate the schooling tendency of an individual using a standardized and repeatable stimulus. We used the model school assay to measure the schooling behavior of threespine sticklebacks (*Gasterosteus aculeatus*), a species in which social grouping behavior has been extensively studied [1], [6]–[11], [13]–[16]. Because the costs and benefits of social grouping vary across ecological conditions, we hypothesized that schooling behavior might differ among populations of sticklebacks that inhabit an open-water marine *vs.* a highly vegetated benthic lake habitat. Specifically, we wanted to answer the following questions: Do sticklebacks from alternative habitats differ in their tendency to school? To what extent are these differences heritable or learned from recent social experience? Can we dissect the stimuli that elicit schooling behavior? The results of these studies not only provide new insight into the mechanisms that underlie the evolution of differences in schooling behavior among fish species, but also into the evolution of differences in sociality among animal groups.

## Results

**Figure 1:**
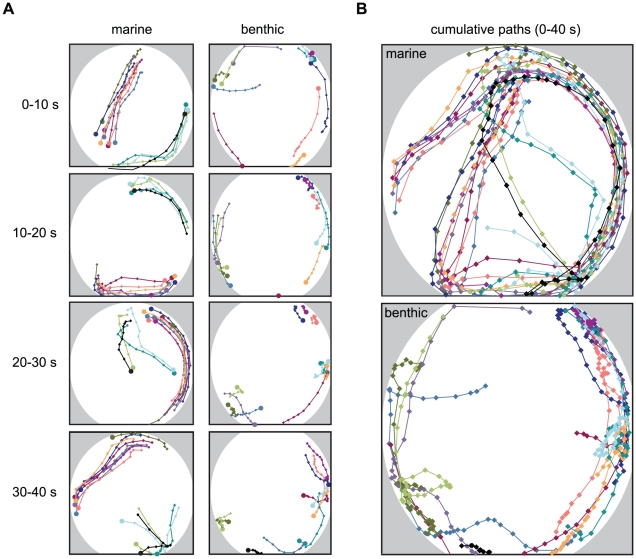
Spontaneous schooling behavior differs between marine and benthic sticklebacks. (A) Twelve individual marine (left column) and benthic (right column) sticklebacks were tracked for 40 seconds in free-swimming groups within a circular tank (indicated by the area in white). Panels show consecutive 10-second increments. Each colored path represents a single individual, and each point represents the position of the individual at a one-second interval. Large circles mark the final position for each time bin and therefore indicate swimming direction. (B) Cumulative tracks for marine (top panel) and benthic (bottom panel) sticklebacks for the entire 40 seconds.

Previous observations of sticklebacks in the wild had noted that marine sticklebacks school strongly while benthic sticklebacks exhibit reduced schooling [17], [18]. To further investigate this behavioral difference, we first made anecdotal observations of free-swimming groups of laboratory-reared sticklebacks from a marine and a benthic population in a controlled laboratory setting. Twelve conspecific sticklebacks from each population were placed into a large circular tank, one population group at a time, and the position of each individual during the first 40 seconds of this interaction was tracked (Fig. 1). These marine and benthic sticklebacks showed different levels of spontaneous schooling behavior. Throughout the tracking period, all marine sticklebacks maintained the same speed as, and parallel alignment with, at least one other fish less than two body lengths away. Eight of the marine sticklebacks schooled together throughout the trial, while the other four started as a single school and divided into two groups of two after approximately 25 seconds (Fig. 1). In contrast, the benthic sticklebacks showed reduced schooling behavior, and many never schooled during the trial period. Two schooling episodes occurred in this group: in the 10–20 second time bin, a group of four fish and a group of two fish schooled together for several seconds (Fig. 1). Both of these cases were transient and lasted less than 10 seconds.

**Figure 2:**
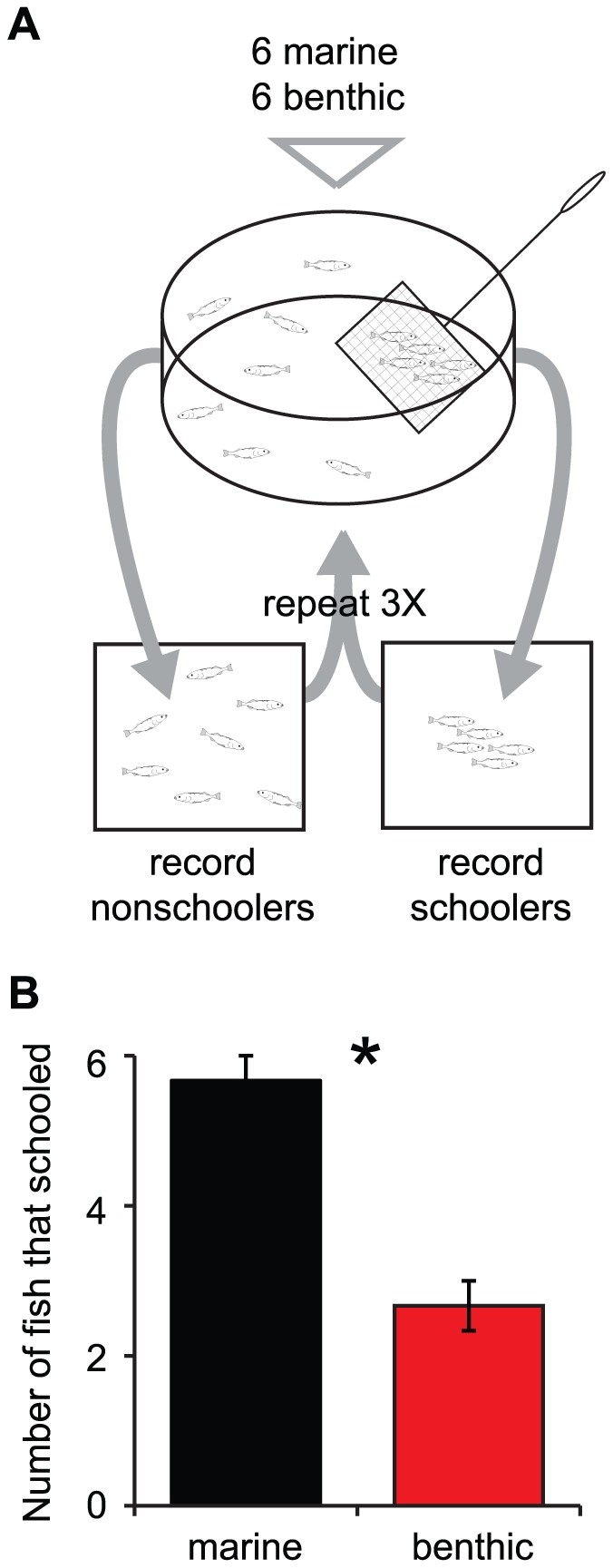
Marine sticklebacks school more than benthic sticklebacks in mixed population schools. (A) Schematic depicting mixed species tournament schooling assay. Six marine and six benthic sticklebacks were placed in a large tank, and all schooling fish were caught in a net. School composition was noted, and schooling fish were marked. Fish were retested two more times, for a total of three rounds. Three independent groups of twelve fish were tested. (B) Marine sticklebacks were found in the school significantly more often than benthic sticklebacks. The mean number of fish (± SEM) from each population that schooled at least once during the assay is shown. *n* = 3 groups of six fish for marine (black bars) and benthic (red bars).

We next asked whether the behavior of benthic and marine sticklebacks might be altered in the presence of heterospecifics. Three independent mixed groups comprised of six marine and six benthic sticklebacks were placed (three times each) into the testing tank (Fig. 2A), and fish were allowed to swim freely. Schooling and nonschooling fish were caught and individual marine and benthic fish were scored as to whether or not they were caught one or more times in a school. Marine sticklebacks were caught with the school significantly more often than benthics (Fig. 2B; *F*_1,5_ = 15.2; *p*<0.02).

**Figure 3:**
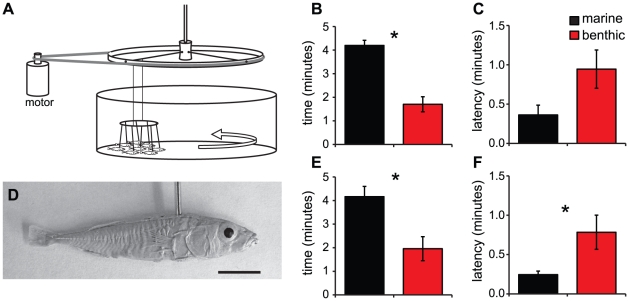
Marine sticklebacks spend more time with the model school than benthic sticklebacks. (A) Schooling behavior was tested by presenting experimental fish with a model school made up of eight polyurethane stickleback casts. An external motor rotated a bicycle wheel above the circular testing tank, causing the model school to “swim” around the tank. (D) Example model stickleback (cast from a benthic×marine F2 hybrid) used in the model school assay. Scale bar = 1 cm. (B,C) results from single population housing conditions (marine: *n* = 19, benthic: *n* = 20); (E,F) results from mixed population housing conditions (marine: *n* = 10, benthic: *n* = 10). (B,E) time spent schooling and (C,F) latency to approach school. All bars represent mean ± SEM. Black bars = marine and red bars = benthic. Asterisks indicate significant comparisons.

**Figure 4:**
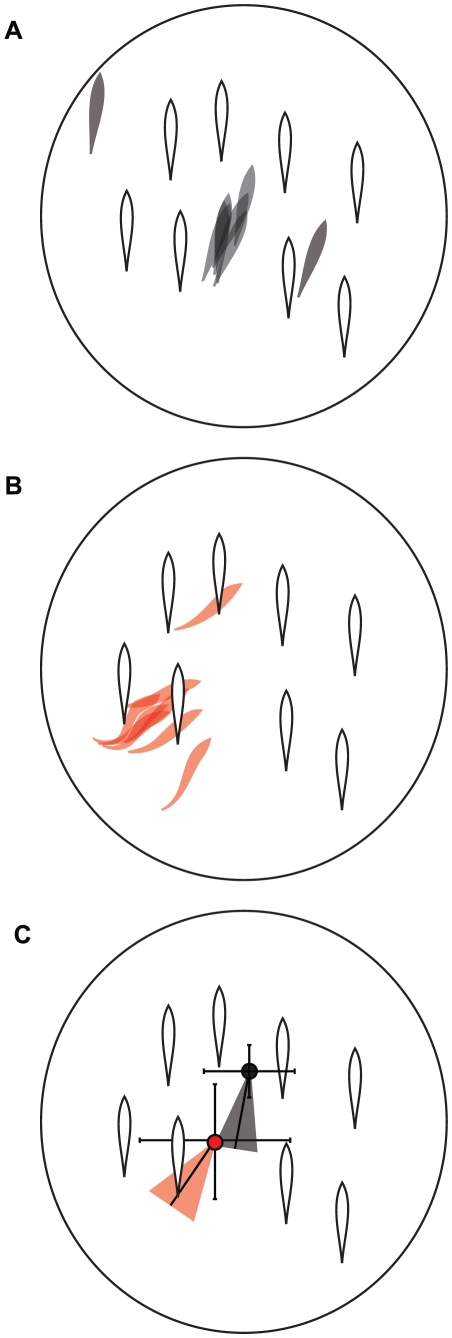
Marine and benthic sticklebacks assume different positions with the model school. (A,B) Silhouettes of schooling marine (A) and benthic (B) sticklebacks traced from video clips during the model school assay. Four frames from each of two fish are shown for each population. These examples were randomly chosen from the subset of frames in which the fish was actively schooling. The position of the model sticklebacks are shown in open silhouettes, test fish are in filled silhouettes. Grey = marine, red = benthic. (C) Summary figure showing the average *x*- and *y*-positions and angle of each population during the model school assay. The *x*- and *y*-position is shown with a filled circle (black = marine, *n* = 19; red = benthic, *n* = 20); error bars represent the standard deviation. The average angle is shown as an angled line, and the standard deviation is represented by a triangle (grey = marine, *n* = 19; red = benthic, *n* = 20). See text for statistical tests.

The above experiments suggested that freely interacting marine and benthic sticklebacks exhibit different schooling tendencies. However, we wanted determine whether the behavior of individuals from these populations varied under identical social circumstances. To do this, we developed the model school assay (Fig. 3). The model school elicited strong schooling behavior from marine sticklebacks, with some fish following the school for the entire 5-minute trial (Fig. 3). The behavior of marine sticklebacks appeared similar to typical schooling behavior exhibited when interacting with groups of live fish: the test fish took parallel positions with the models and occasionally changed their position amongst the models (Video S1; Fig. 4A). However, benthic fish showed a significantly different response to this stimulus, and most fish did not follow the models for long periods of time or maintain a parallel position with the school (Video S1; Fig. 4B).

To quantify the difference between the populations, we measured several aspects of behavior: total time with the school and latency to initially join the school, as well as the average *x*- and *y*-positions and body angle when swimming with the model school. Marine sticklebacks spent a significantly greater amount of time with the school than benthic sticklebacks (Fig. 3B; *F*_1,36_ = 20.9, *p*<0.04) and also showed a shorter latency to initially join the school, although this did not reach statistical significance (Fig. 3C; *F*_1,36_ = 4.6, *p* = 0.16). Marine and benthic sticklebacks also assumed different positions when following the school. Marine sticklebacks showed a more parallel position with the models than did benthic sticklebacks (Fig. 4C; marine = 83±2°; benthic = 54±4°, with 90° representing a parallel position with the models; *F*_1,36_ = 37.8, *p*<0.03). The average *x*- and *y*-positions also differed between populations, but did not meet statistical significance (Fig. 4C; *x*-position: *F*_1,36_ = 6.6, *p* = 0.12; *y*-position: *F*_1,36_ = 16.0, *p* = 0.057).

All fish in this experiment were reared in the laboratory without parental contribution, suggesting that this behavioral difference is heritable. However, this could be an innate difference that is reinforced by exposure to the similar social behavior of siblings. In order to examine the plasticity of this behavior, we also tested sticklebacks that were housed in mixed population social groups for two months before testing. We found that the behavioral difference between populations was maintained in fish that were housed in mixed population groups. As with fish housed in single population groups, marine sticklebacks from mixed population housing spent more time with the school than benthic sticklebacks (Fig. 3E; *F*_1,19_ = 10.9, *p*<0.004), and they also joined the school with shorter latencies (Fig. 3F; *F*_1,19_ = 5.9, *p*<0.03).

**Figure 5:**
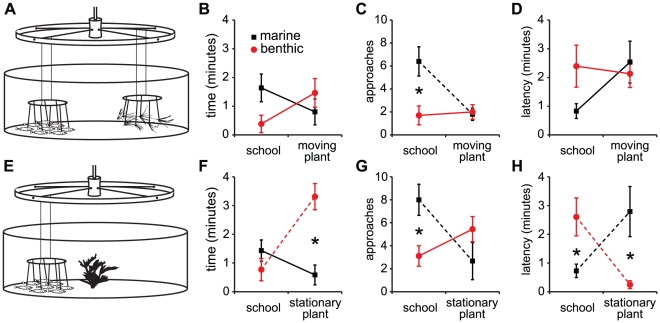
Marine and benthic sticklebacks display different preferences for social and non-social stimuli. (A,E) Schematic diagrams showing assays used for testing preference for the model school or plant stimuli using either (A) moving (marine: *n* = 10, benthic: *n* = 10) or (E) stationary plant (marine: *n* = 9, benthic: *n* = 9) choices. (B,C,D) results from moving plant trials and (F,G,H) results from stationary plant trials. (B,F) time spent with school or plant; (C,G) number of approaches to school or plant; (D,H) latency to approach school or plant. Black symbols and lines represent marines; red represent benthics. A dashed line indicates a significant pairwise comparison between plant and school for a single population. An asterisk indicates a significant pairwise comparison between benthic and marine for a single stimulus.

We next sought to dissect some of the stimuli that contribute to the propensity to follow the model school. First, we wanted to test the possibility that fish were not actually “schooling” with the model school, but rather seeking shelter with the only form of shelter available in the tank (the moving model school). This was motivated by our observation that the benthic sticklebacks that do follow the school typically do not take parallel positions with the models and instead often appear as though they are “hiding” underneath the models (Fig. 4), whereas the marines exhibit obvious features of schooling behavior with the model school (Video S1, Fig. 4). Second, we wanted to determine whether joining the model school results from a tendency to indiscriminately follow any moving stimulus. It is known that the optomotor response causes fish to follow movement and can induce schooling-like behaviors [19], [20], and sticklebacks exhibit an optomotor response [21]–[23]. To test these possibilities, we modified the model school assay to include an alternative choice of a non-social shelter (an artificial plant) in the assay tank. We configured the plant in two ways: in the “moving plant” assay, the plant rotated opposite the model school; in the “stationary plant” assay, the plant was fixed at the center of the tank (Fig. 5A,E). For each trial, we made six measurements: time spent with plant and school, latency to approach the plant and school, and the number of approaches to the plant and school.

When presented with a moving plant as an alternative to the model school, marine and benthic sticklebacks exhibited significantly different behavior. Marine sticklebacks made more approaches to the school when compared to benthic sticklebacks (Fig. 5C; *F*_1,18_ = 9.1, *p*<0.007). This was driven by a significant preference for the school among marine sticklebacks, as they made significantly more approaches to the school than the plant (significant post-hoc comparison indicated by dashed line in Fig. 5C). There was a similar statistical trend for latency to approach the stimuli, although this did not meet statistical significance (Fig. 5D; *F*_1,18_ = 4.3, *p* = 0.053). In contrast to marines, benthic sticklebacks appeared indifferent to the school and plant, showing similarly low levels of behavior toward both stimuli (Fig. 5B–D).

The choice of a stable plant *vs.* the moving model school also led to significantly different preferences between marine and benthic fish (asterisks in Fig. 5F–H; time: *F*_1,18_ = 10.8, *p*<0.005; number of approaches: *F*_1,18_ = 10.3, *p*<0.006; latency: *F*_1,18_ = 13.5, *p*<0.002). Marine sticklebacks demonstrated a significant preference for the school, as indicated by both excess approaches and a shorter latency to approach the model school (significant post-hoc comparisons indicated by dashed black lines in Fig. 5G,H). By contrast, benthic sticklebacks demonstrated a significant preference for the stationary plant, as indicated by excess time spent with and a reduced latency to approach the plant (significant post-hoc comparisons indicated by dashed red lines in Fig. 5F,H).

Marines spent less time schooling overall in the stimulus preference assays when compared with the model school alone (no choice, average time schooling = 4.2±0.2 min; moving plant assay, average time with both stimuli = 2.4±0.5 min; stationary plant assay, average time with both stimuli = 2±0.4 min).

## Discussion

In this study we investigated variation in schooling behavior between two populations of stickleback from different habitats. Our tracking data show that free-swimming groups of laboratory-raised marine and benthic sticklebacks exhibit differences in schooling behavior. Marine sticklebacks spontaneously formed cohesive schools whereas only a subset of benthic sticklebacks formed small, transient schools. These differences persisted in mixed population groups: individual marine sticklebacks still schooled strongly in the presence of nonschoolers, and individual benthic sticklebacks did not school strongly even in the presence of strong schoolers.

In order to further investigate the mechanistic basis for this divergent behavior, we developed an assay to test the schooling behavior of individuals. Using the model school assay, we were able to reliably elicit schooling behavior in the laboratory under repeatable stimulus conditions. Marine sticklebacks showed clear indications of schooling behavior in this assay: they exhibited polarized, coordinated movement with the model school (Video S1; Fig. 4) [3], [4]. We were able to use this assay to conclusively demonstrate that individual marine sticklebacks have a stronger tendency to school than benthic sticklebacks in the laboratory. We have also shown that when benthics do associate with the school, they assume different positions with the models than do marines. Thus, our positional analysis demonstrates that our assay can differentiate between schooling (marine sticklebacks) and shoaling, as indicated by following the model school in a non-polarized way (benthic sticklebacks). Although differences in schooling tendencies among populations of fish have been previously reported [24]–[27], our study is novel in that the model school assay allows us to assess the schooling behavior of individuals without the confounding effects of the behavior of other individuals in the school.

Because the fish we tested were reared in the laboratory, without the influence of parental care, our data suggest that the difference in schooling tendency between marine and benthic sticklebacks is heritable. This is consistent with previous work demonstrating that differences in schooling tendency among populations of guppies and minnows have a genetic basis [24], [28], [29]. However, it has been shown that early experience with predators or conspecifics can influence the extent to which fish engage in social behavior [13], [29]. In order to assess the effect of experience with conspecifics on schooling behavior, we also housed some marine and benthic sticklebacks in mixed population groups. If experience with schooling was important for benthic fish to show schooling, their schooling tendency should have increased following exposure to the schooling marine sticklebacks. However, our results show that the behavior of the benthics was still significantly different from that of the marines following mixed population housing. This indicates that the difference in schooling behavior between marine and benthic sticklebacks is not only heritable, but is also robust to exposure to the behavior of conspecifics. Nonetheless, to fully explore the plasticity of this behavior, it will be important to test fish that have been reared in mixed population groups for their entire life or that have been exposed to predators.

In addition to providing an opportunity to investigate the genetic and environmental influences on schooling behavior, the model school assay has already enabled novel insights into the mechanisms of schooling behavior. For example, schooling behavior is typically studied as an emergent property of groups of fish, yet it results from the behavior of individuals [30]–[32]. Although experimental and theoretical work has demonstrated that the presence of an informed individual can “seed” collective movement [5], [7], it is not known whether differences in the propensity to school across populations or species are due to the presence of such key individuals or to a shared tendency of all individuals in a school. Because the model school assay allowed us to test individual schooling behavior outside the context of a live social group, we were able to demonstrate that differences in schooling across stickleback populations are strongly influenced by the shared behavioral predispositions of individuals in a school (see also [33]).

What is the basis for differing tendencies to join the model school? There are several possible explanations for the divergent responses of benthic and marine sticklebacks. First, the speed and arrangement of the model school was based on a Japanese marine school, thus, it could be argued that the benthic sticklebacks might school if given a more “benthic-like” schooling stimulus. However, their reduced schooling tendency in the model school assay strongly reflects their behavior in free-swimming groups. Furthermore, some benthic sticklebacks do follow the school, albeit for significantly less time, suggesting that the difference in association is not due to an inability to detect and join the school, or a “fear” of the school. Because these populations show divergent behavior under identical social conditions, we favor two additional (and not mutually exclusive) explanations for their divergent responses to the model school. Benthic and marine sticklebacks might have sensory differences that lead to differences in stimulus recognition, and/or differences in their motivation to follow the school.

Using our assay, we were able to begin to explore the stimuli that are important for the choice to associate with the school, and whether these stimuli differ among populations. In particular, we investigated whether a general tendency to follow movement or to seek shelter contributed to joining the model school. Our results suggest the nature of the stimulus and not simply the fact that the stimulus is moving drives association with the model school. Moreover, it appears that the two populations utilize different stimuli in their decisions to school. Marines show a preference for the social stimulus of the model school over an artificial plant, whether moving or stationary. By contrast, when benthics follow the model school, it appears to result from a drive to seek shelter. When both stimuli are moving, benthics are indifferent to following the plant or the model school, suggesting that both types of shelter are equally (un)-attractive. However, when presented with a stable shelter, benthics showed a strong preference for associating with the non-moving plant. In concert with this idea, analysis of body position with the model school revealed that the typical “schooling” position of benthic sticklebacks was consistent with the fish “hiding” beneath the models.

Like many forms of social grouping, schooling is frequently viewed as a form of protection against predators, particularly for animals that lack other shelter [2]. One mechanism underlying the evolutionary loss of schooling behavior could be a dissociation of the need to find shelter from the drive to behave socially. In concert with this idea, our results suggest that benthic sticklebacks, which have abundant shelter options in their natural habitat [17], do show an equal or even enhanced preference for non-social shelter stimuli. This suggests that the reduced schooling behavior in benthic sticklebacks might be driven in part by an enhanced preference for non-social, non-moving shelter options. This novel observation may shed light on the alterative neural processes that have led to the evolution of different schooling behaviors, and more generally to varying levels of sociality observed among other animals.

With simple adjustments to the number or features of models, the size and spacing of the school, or the sensory environment, the model school assay that we have developed could be used to further dissect the key external and internal stimuli required to elicit schooling behavior. The combination of a rigorous but adjustable assay with sophisticated behavioral analysis will enable long-awaited empirical dissection of the genetic and neural mechanisms that underlie schooling behavior in sticklebacks, as well as other fish species.

## Materials and Methods

### Ethics statement

All animal work was conducted according to relevant national and international guidelines. This work was approved by the Fred Hutchinson Cancer Research Center Institutional Animal Care and Use Committee (FHCRC IACUC protocol #1575).

### Stickleback populations

We compared the schooling behavior of two stickleback populations, a marine population from the Pacific Ocean in Japan and a freshwater benthic population from Paxton Lake in Canada. The Japanese marine population used in the present study was originally caught at the Bekanbeushi River in Akkeshi on Hokkaido Island, Japan [34]. Like many marine sticklebacks, this population is anadromous and spends the majority of the year in the ocean, migrating into stream and lake habitats to breed. In contrast, Paxton benthic sticklebacks occupy the heavily vegetated littoral zone of Paxton Lake, located on Texada Island in British Columbia, Canada [17], [35]. Paxton Lake is not connected to the ocean, and this population has been isolated from marine ancestors since the last ice age, approximately 12,000 years [36].

All Japanese marine and Paxton benthic sticklebacks used in these experiments were the laboratory-reared progeny of either wild-caught or first-generation, lab-raised parents. Following *in vitro* fertilization, fish were raised without parental care in 110 liter aquarium tanks (75×46×30 cm). All fish were kept in stickleback tank water [0.35% saltwater (Instant Ocean, Aquarium Systems, Mentor, OH, USA) buffered with 44 mg/l sodium bicarbonate (Sigma Aldrich, St. Louis, MO, USA)] on a summer lighting schedule (16 hours light/8 hours dark) at approximately 15.5°C. Fish were fed live *Artemia* nauplii twice daily.

### Observation of group schooling behavior

We first made anecdotal observations of the spontaneous schooling behavior of groups of laboratory-reared marine and benthic sticklebacks while swimming freely in a large tank. The assay was conducted in a large (102 cm diameter, 62 cm depth) “koi show bowl”. The tank was filled to a depth of 8 cm with stickleback tank water (see above). The assay tank was illuminated using two 60 W incandescent lamps as well as full spectrum fluorescent room lighting. Twelve marine or twelve benthic fish were removed from their home tanks and placed in a pre-trial acclimation chamber (29×18×12.5 cm) for five minutes. After this acclimation period, all twelve fish were transferred at once to the tank, and their behavior was videotaped from above for fifteen minutes. The position of each fish was tracked at one-second intervals for the first 40 seconds of the trial using StickleTrack software (Physion Consulting, Boston, MA, USA). All fish from each population were from a single family. Benthics were 18 months old with an average standard length (± S.E.M.) of 4.3±0.3 cm and marines were 10 months old with an average standard length (± S.E.M.) of 3.2±0.2 cm. All fish were prereproductive.

We also scored the schooling tendency of freely swimming fish in mixed population groups. At the start of each trial, six marine and six benthic fish (from the same families used above) were removed from their home tanks and placed together in a pre-trial acclimation chamber for five minutes. Marines and benthics can be distinguished by morphology, so there was no need to mark fish from the two populations differently. After this acclimation period, all twelve fish were transferred at once to the testing tank. Fish were allowed to swim freely while an experimenter observed. After two minutes, the experimenter waited for the schooling and nonschooling fish to move a sufficient distance apart from one another and then caught all schooling fish with two nets. The schooling and nonschooling fish were placed in two separate tanks. To keep track of individual fish in subsequent rounds of mixed population schooling, all schooling fish were given unique marks using discrete clips of the dorsal or pelvic spines, or the caudal fin. All twelve fish were returned to the pre-trial isolation chamber for five minutes, and testing was repeated two additional times. The entire assay was repeated for a total of three independent groups of six marine and six benthic sticklebacks.

### Model school assay construction

The “model school assay” makes use of an artificial stimulus school in order to elicit schooling behavior from individuals using identical conditions across trials. To simulate a school of sticklebacks, we created eight polyurethane model sticklebacks, as previously described [37]. There is a long history of using models to elicit social behaviors in sticklebacks [38], including movable models [6], [7], [39], [40]. Briefly, a mold was cast from a Paxton benthic×Japanese marine F2 hybrid stickleback to create a model that was intermediate in phenotype between the two populations. Models were made from quick-curing polyurethane resin tinted with grey pigment (TAP Plastics, Stockton, CA, USA). Black acrylic paint was applied to the models' eyes to simulate a more realistic appearance. Models were 5.0 cm long. To construct the model school, we used plastic-coated steel wires that were pre-embedded into each model to attach eight stickleback models to a ring of the same plastic-coated steel wire (light metallic aqua colored wire; product #123115; The Hillman Group, Cincinnati, OH, USA). This resulted in a rigid school “mobile” in which the models were individually positioned to mimic the angles and spacing taken from a representative video frame of the school of Japanese marine sticklebacks that was videotaped for the observation of group schooling behavior (see previous section).

Schooling trials were conducted in a custom-built circular white acrylic tank (61 cm diameter, 26 cm depth; TAP Plastics, Stockton, CA, USA) filled with 8 cm of stickleback tank water (see above). A modified bicycle wheel and hub were suspended above the bottom of the assay tank. The bicycle wheel was connected to an external adjustable-speed motor via a rubber belt that allowed the motor to rotate the wheel above the tank. A metal arm was built onto the lower rim of the bicycle hub in order to hang the model school into the tank. Individual models were positioned between 2.5 and 5.0 cm above the bottom of the tank. When the motor was turned on, the models moved in a circular rotation around the tank at a rate of 5.5 rotations per minute, matching the speed calculated from representative video frames of the school of Japanese marine sticklebacks videotaped for the observation of group schooling behavior (see above). The assay tank was illuminated with indirect lighting from a 60 W incandescent lamp. Both rotation speed and model position were invariant across trials.

### Model school assay trials

Before each trial, sticklebacks were taken from their home tanks and placed in individual isolation chambers (12×12×8 cm) behind a closed curtain for at least two hours, in order to standardize the experience of each fish leading up to its trial. Fish were fed *Artemia* nauplii in their home tanks between 2.5 and 8 hours before their trial; we found no difference in behavior due to testing order (and therefore satiation). At the start of each trial, an individual was moved in its isolation chamber to the testing area and transferred with a net to the assay tank for testing. Once animals were placed in the assay tank a blackout curtain surrounding the assay tank was closed for the remainder of the trial. Each trial began with a five-minute acclimation period in which the model school was not moving. The trial fish was free to explore the tank during this period. At the end of the acclimation period, the motor was turned on remotely, and the model school was rotated around the tank for five minutes.

#### Model School Experiment 1: Population differences

We first compared the behavior of twenty marine and twenty benthic sticklebacks in the model school assay. Animals were reared for six months in high-density tanks and were then split into tanks with 20 siblings and reared for an additional two months. We tested ten fish from each of two independent families for each population. None of these families were used in the observations of group schooling behavior, and none of these individuals were tested in any other experiment. One marine individual was excluded from the population schooling comparison because it was a reproductive male displaying territorial behavior, leaving 19 marine fish. Average standard lengths (± S.E.M.) of fish were: benthics, 5.1±0.1 cm; marines, 4.9±0.1 cm.

#### Model School Experiment 2: Effect of social experience

To determine the effect of social experience on schooling behavior, we exposed fish to mixed population groups. At six months of age, fish from one of the marine families and one of the benthic families used in experiment 1 were combined in two tanks; each tank contained ten marine and ten benthic individuals. Observations showed that fish did not segregate by population and were typically found intermingling in the tanks. After two months of mixed population housing, we tested five fish of each population from each tank in the model school assay, yielding ten marine and ten benthic individuals. Average standard lengths (± S.E.M.) of fish were: benthics, 4.9±0.1 cm; marines, 5.0±0.1 cm.

#### Model School Experiment 3: Stimulus preference: moving plant

To explore the stimuli required to induce schooling, we presented fish with a choice between the model school and either a moving or stable plant as shelter. We constructed an artificial plant based on images of *Chara tomentosa* algae, which is a source of stickleback shelter in Paxton Lake [17]. For the moving plant experiment, the artificial plant was constructed using green yarn embedded with moldable wire. Several stalks were then attached to a ring of plastic-coated wire, mimicking the shape and volume of the model school. Ten fish from a single family per population were tested in the moving plant trials; these fish were from new families not previously tested in the above experiments. The benthics were 11 months of age with an average standard length (± S.E.M.) of 5.0±0.1 cm, and the marines were 8 months of age with an average standard length of 4.9±0.1 cm.

#### Model School Experiment 4: Stimulus preference: stationary plant

For the stationary plant experiments, we constructed a less rigid plant out of strips of black plastic trash bag material. The resulting buoyant stalks were attached to a weighted base fixed at the center of the assay tank. Nine fish per population were tested in the stationary plant trials; these fish were siblings of and the same age as the fish in experiment 3. Average standard lengths (± S.E.M.) of fish were: benthics, 4.9±0.1 cm; marines, 4.9±0.1 cm.

### Video analysis

Model school trials were recorded in 1080i format using a SONY HC9 digital camcorder (SONY, San Diego, CA, USA). Videos were digitized using iMovie software (Apple, Inc., Cupertino, CA, USA) and were tracked using Minutes tracking software (Physion Consulting, Boston, MA, USA). We scored schooling behavior using the following criteria: a schooling bout began when the experimental stickleback swam in the same direction as the school (counter-clockwise) within one body length of the closest model. Maintaining exact parallel alignment with the models was not required. If a fish stopped swimming, changed direction, or swam beyond one body length from a model, the schooling bout was ended. Equivalent criteria were used for tracking association with artificial plants. We then calculated the total time spent with each stimulus and latency to initially approach the stimulus. Increased time schooling and decreased latency indicate a greater attraction to the stimulus. Note that these measures are somewhat interdependent: a fish with a long latency cannot have spent much time schooling. For the stimulus preference trials, we also measured the number of approaches to the stimulus; more approaches indicate a stronger preference.

For analysis of the angle and *x*- and *y*-positions of the test fish with the models, single video frames were extracted from video files using QuickTime 7.0 Pro (Apple, Inc., Cupertino, CA, USA). We sampled a single frame per rotation; each of these frames was taken when the model school was at a predetermined position in the tank. Although there were 26 possible frames for each fish, we only analyzed frames when the fish was within one body length of the models and swimming in the same direction as the model school. Thus, fish had variable numbers of frames for analysis, depending on how much time each individual spent near the school. Files were imported into ImageJ (http://rsbweb.nih.gov/ij/index.html) for analysis. To calculate the average angle of a fish relative to the models, the angle of the straight portion of the body of the test fish was measured in each frame: 90° represents a parallel alignment with the school and 0° indicates that the fish was facing toward the right. To calculate the average position for each fish, the *x*- and *y*-positions of the head of each model and the test fish were recorded in each frame.

### Statistics

Behavioral measurements for marine and benthic sticklebacks in model school experiment 1 were compared using population as a fixed factor in a general linear model in R Statistical Software (http://www.r-project.org). To avoid pseudoreplication, we included individual nested within family as a random factor in the model. The remaining experiments were conducted on a single family per population, so it was not necessary to include family in the model. Behavioral measurements from model school experiment 2 were compared by testing the effect of population using an ANOVA in SPSS 13.0 software (SPSS, Inc., Chicago, IL, USA). For the model school stimulus preference experiments 3 and 4, we used a general linear model in SPSS to test for an interaction between the within-subjects factor “stimulus” (plant or school) and the between-subjects factor “population” (marine or benthic). When a significant interaction was found, we used least significant difference (LSD) post-hoc tests to identify significant contrasts (effect of population within each stimulus condition; choice of plant or school within each population).

## Supporting Information

Video S1 **Marine (left) and benthic lake (right) sticklebacks display behavioral differences in the model school assay.**
(MOV)
